# Detection of Extended-Spectrum *β*-Lactamases (ESBL) Producing Enterobacteriaceae from Fish Trapped in the Lagoon Area of Bizerte, Tunisia

**DOI:** 10.1155/2020/7132812

**Published:** 2020-06-09

**Authors:** Bilel Hassen, Ahlem Jouini, Monia Elbour, Safa Hamrouni, Abderrazek Maaroufi

**Affiliations:** ^1^Institute of Veterinary Research of Tunisia, University Tunis El Manar, 20 rue Jebel Lakhdhar, Bab Saadoun, Tunis 1006, Tunisia; ^2^Laboratory of Epidemiology and Veterinary Microbiology, Group of Bacteriology and Biotechnology Development, Institute Pasteur of Tunis, University of Tunis El Manar, Tunisia; ^3^Marine Laboratory, The National Institute of Sciences and Technology of the Sea, University of Carthage, 2025 Salammbô, Tunisia

## Abstract

Extended-spectrum *β*-lactamase and their molecular mechanism in *Enterobacteriaceae* were analyzed in 126 fish samples of 9 various wild species, living in the lagoon of Bizerte in Tunisia. Fifty-nine (59) Gram-negative strains were isolated and identified as *Escherichia coli* (*n* = 24), *Klebsiella pneumonia* (*n* = 21), *Citrobacter freundii* (*n* = 8), and *Shigella boydii* (*n* = 6). Forty-seven ESBL producers were identified using the synergic test. *β*-Lactamase genes detected were *bla*_CTX-M-1_ (*E. coli*/15; *K. pneumonia*/8; *C. freundii*/1; *Sh. boydii*/1), *bla*_CTX-M-1_+ *bla*_OXA-1_ (*E. coli*/4; *K. pneumonia*/3), *bla*_CTX-M-1_+ *bla*_TEM-1-a_ (*K. pneumonia*/2), *bla*_CTX-M-15_+ *bla*_TEM-1-a_ (*K. pneumonia*/1; *Sh. boydii*/1), *bla*_CTX-M-15_+ *bla*_OXA-1_ (*K. pneumonia*/1), *bla*_CTX-M-15_ (*E. coli*/3; *K. pneumonia*/1; *Sh. boydii*/3), and *bla*_CTX-M-9_ (*C. freundii*/3). Most strains (84.7%) showed a multiresistant phenotype. *qnrA* and *qnrB* genes were identified in six *E. coli* and in ten *E. coli*+one *K. pneumonia* isolates, respectively. The resistance to tetracycline and sulfonamide was conferred by the *tet* and *sul* genes. Characterization of phylogenic groups in *E. coli* isolates revealed phylogroups D (*n* = 20 strains), B2 (*n* = 2), and A (*n* = 2). The studied virulence factor showed prevalence of *fimA* genes in 9 *E. coli* isolates (37.5%). Similarly, no strain revealed the three other virulence factors tested (*eae*, *aer*, and *cnf1*). Our findings confirmed that the lagoons of Bizerte may be a reservoir of multidrug resistance/ESBL-producing *Enterobacteriaceae*. This could lead to indisputable impacts on human and animal health, through the food chain.

## 1. Introduction

The increasing rates of land-based anthropogenic pollution in marine ecosystems have become an important factor that promotes the emergence of multidrug-resistant (MDR) bacteria in aquatic animals [1–3]. The rapid dissemination of extended-spectrum *β*-lactamase-producing *Enterobacteriaceae* in marine coastal ecosystems is worrisome because enterobacteria species (mostly *Escherichia coli*) are commensal bacteria in the gut microbiota of fishes [4, 5].

Fish living in the natural environment harbored pathogenic *Enterobacteriaceae* [6–8]. Therefore, fish are considered as a potential vehicle of foodborne bacterial infections, which may present a threat for the human public health. Contamination of fish with MDR bacteria could demonstrate the risk of the persistence of these bacteria in the fish gut flora and explain possible human gut contamination [9]. Besides, significant antibiotics are excreted unaltered or as metabolites (up to 75%), which present a major source of antibiotic input into the aquatic environment. It is estimated that 49% of marine ecosystems worldwide are strongly affected by some anthropogenic factors of stress with significant and serious economic implications [10, 11]. Many of these compounds can now be detected easily in water resources.

Human survival and well-being depend on different services of the marine ecosystem (such as fishing) and, therefore, on the conservation and the best management of the ecosystems. Research on marine or lagoon ecosystem services has grown exponentially during the last decade, for better marine risk assessments. This pollution from a variety of sources (urban, agricultural, and industrial) contributed to altering the communities of different living beings that are prevailing in the marine and lagoon environment and indirectly presenting risks for human health [9, 12].

If mismanaged, multiple anthropogenic impacts on marine ecosystems might also affect coastal fisheries and aquaculture. The wide use of antimicrobial agents in clinical settings, veterinary medicine, livestock industries, and aquaculture has led to a large-scale dissemination of antibiotic-resistant bacteria in many environments [7, 8, 13, 14].

For enterobacteria, the problem of antibiotic resistance is essentially related to the broad-spectrum family of cephalosporin. Such resistance is usually due to the production of extended-spectrum *β*-lactamases (ESBLs) or cephalosporinase plasmid (*pAmpC*). The producing ESBL or *pAmpC* strains are largely isolated in hospital settings, animals, and food products and from different aquatic environments (such as lakes, rivers, and urban runoff). The CTX-M gene is the most common ESBL gene detected [15].

Resistance genes may be transferred between bacteria via mobile genetic elements [16]. One such mobile element called integrons that mediate the integration of resistance genes may also be involved, resulting in the development of multidrug-resistant bacteria [17].

Thus, the lagoon of Bizerte could be a site of choice allowing studies of the important triangular thematic aspect of “pollution-aquatic ecosystems-biotic component,” by the national and international scientific communities [18–20]. The lagoon of Bizerte receives various discharges through urban and industrial wastewater and agricultural runoff [21, 22].

The main aim of this work was to detect, isolate, and identify species multiresistant to antibiotics and producing ESBL enterobacteria (ESBL-Eb), from the intestines and gills of some species of wild fish, trapped in the lagoon of Bizerte. In the first part, we have studied the mechanisms of resistance in these enterobacteria isolates phenotypically and genetically. In the second part, we investigated important virulence factors involved in these isolates.

## 2. Materials and Methods

### 2.1. Sites of Fish Sampling

**Figure 1:**
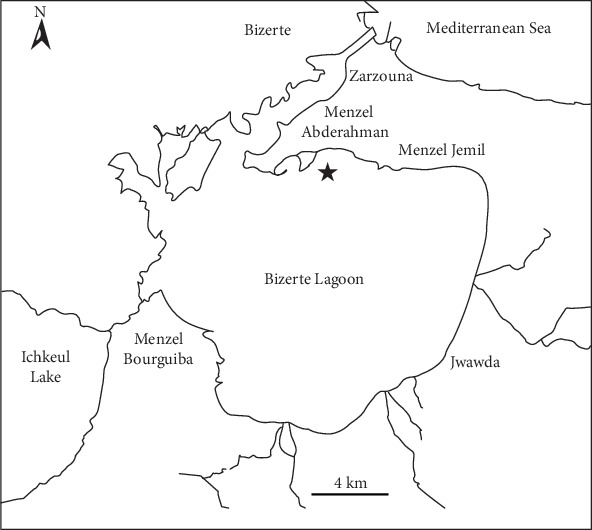
Map of Bizerte lagoon (Northern Tunisia). The black full star in the northern part of the lagoon shows the sampling site location.

The isolation of enterobacteria strains was performed by analyzing 126 samples of 9 wild species of fish trapped during the second half of the year 2016, in the surroundings of Menzel Abdurrahman port (north coast of Bizerte lagoon) (Table 1, Figure 1). The lagoon of Bizerte is an isodiametric saltwater body, located between latitude 37° 8′ and 37° 16′ N and longitude 9° 46′ and 9° 56′ E, in the north of Tunisia. It covers approximately 150 km^2^ and has an average depth of 8 m. It communicates with the Mediterranean Sea by a channel of 8.5 km in length and is connected to the Lake of Ichkeul by the river of Tinja. It represents an economically important body of water due to a variety of fishing and aquaculture activities. These fish samples were transported aseptically to the laboratory in a refrigerated cooler at +4°C and analyzed within the 24 h following their collection. The samples of fish tissues were primarily experimented for their intestinal tract and gills.

### 2.2. Bacteria Isolation

After dissecting the sampled fish, the gills and the stomach contents were separately inoculated into 225 mL of buffered peptone water, pH = 7. After incubation under shaking for 24 h at 37°C, dilutions in sterilized physiological water were performed, and a volume of 1 mL was seeded on MacConkey agar growth medium supplemented with 2 *μ*g/mL of cefotaxime (CTX) for enterobacteria recovery. All the plates were incubated at 37°C for 24 h.

From each inoculate sample and based on colony size and morphology, a maximum of five suspected *Enterobacteriaceae* colonies were selected for *E. coli* isolate identification by biochemical tests and specific PCR amplification of the *uidA* gene [23]. Identification of other *Enterobacteriaceae* was realized by PCR amplification and sequencing of 16S rRNA genes [24]. *E. coli* strain (ATCC 25,922) was used as the control.

### 2.3. Antibiotic Susceptibility Testing

Susceptibility to 16 antimicrobial agents was performed using the disk diffusion method, following the Clinical and Laboratory Standard Institute (CLSI) recommendations [25]. The following antimicrobial agents were tested (concentration in *μ*g): ampicillin (10 *μ*g), cefotaxime (30 *μ*g), cefoxitin (30 *μ*g), ceftazidime (30 *μ*g), chloramphenicol (30 *μ*g), ciprofloxacin (5 *μ*g), gentamycin (10 *μ*g), imipenem (10 *μ*g), nalidixic acid (30 *μ*g), streptomycin (10 *μ*g), sulfamethoxazole (300 *μ*g), tetracycline (30 *μ*g), ticarcillin (75 *μ*g), tobramycin (10 *μ*g), trimethoprim (5 *μ*g), and trimethoprim-sulfamethoxazole (1.25 + 23.75 *μ*g). A screening test for the detection of ESBLs was carried out by the double-disc synergy test (DDST), according to the CLSI criteria [25].

### 2.4. Antibiotic Resistance Gene Characterization

The genes encoding TEM, SHV, OXA, and CTX-M *β*-lactamases were analyzed by PCR and sequencing in all ESBL-Eb [23]. The nucleotides and their amino acid sequences were compared with those deposited in the GenBank database and those reported in the website http://www.lahey.org/Studies, to confirm the specific *β*-lactamase genes. Genes encoding resistance to tetracycline (*tet A* and *tet B*), quinolone (aac[6′]-Ib, *qnrA* and *qnrB*), and sulfonamide (*sul* 1 and *sul* 2) were analyzed by PCR (and sequencing sometimes) [26].

### 2.5. Phylogenetic Groups and Virulence Factors

Identification of the major phylogenetic groups of *E. coli* isolates was determined by PCR, using a combination of three genes (*chuA*, *yjaA*, and *tSpeA.C2*) [27].

*E. coli* strains were screened for the following virulence factors: *eae* that codes for intimin, *aer* for aerobactin, *cnf1* for cytotoxic necrotizing factors, and *fimA* for fimbriae of type I genes [28].

## 3. Results

### 3.1. Identification of Isolates

Enterobacteria (*n* = 59) were isolated from 126 wild fish sampled from the Bizerte lagoon. All recovered isolates were cefotaxime resistant, and the number of isolates from the gills (*n* = 35) was slightly higher when compared to those from the viscera and the stomach contents (*n* = 24) (Table 1). Biochemical and molecular identification showed that the 24 isolates were assigned to *E. coli* species harboring the specific gene *iudA*. Analysis of the sequences of the 16S rRNA genes of the 36 remaining isolates allowed the characterization of three enterobacteria species: *Klebsiella pneumonia* [*K. pneumonia*] (*n* = 21), *Citrobacter freundii* [*C. freundii*] (*n* = 8), and *Shigella boydii* [*Sh. boydii*] (*n* = 6).

The rate of enterobacteria strain isolation showed a high ratio of distribution over the fish species in the Bizerte lagoon. However, we noticed a low ratio of segregation for the fish species of *Lithognathus mormyrus*, *Pomatomus saltatrix*, and *Solea solea*, with a ratio close to zero (Table 1).

### 3.2. Antimicrobial Resistance Phenotypes

The highest resistance frequency among the 59 cefotaxime-resistant strains was registered for the family of *β*-lactam antibiotics. The respective antibiotic resistance percentages were of 100% for ampicillin, 96.3% for ticarcillin, 11.9% for amoxicillin/clavulanic acid, 40.7% for ceftazidime, 24% for cefoxitin, 74.6% for ertapenem, and 5.1% for imipenem.

This highest resistance frequency to *β*-lactam was followed by an important resistance frequency of 71.2% for the sulfonamides. For non-*β*-lactam antibiotics, such isolate resistance frequencies were as follows for tobramycin (47.5%), gentamicin (40.7%), nalidixic acid (37.3%), tetracycline (37.3%), ciprofloxacin (3.4%), and chloramphenicol (1.7%). None of the strains was resistant to trimethoprim/sulfamethoxazole. Fifty (84.7%) out of the 59 isolates showed multiresistant phenotypes, including resistance to at least three families of antimicrobial agents. A higher multiresistance was noticed especially for the isolates from the gills than from the viscera and the stomach (58% and 42% of the isolates, respectively). Detection of ESBL isolates by the double-disc synergy test (DDST) revealed that 47 cefotaxime-resistant strains were ESBL-Eb-producing ones and belonged further to the following four species: *E. coli* (*n* = 22), *Klebsiella pneumonia* (*n* = 16), *Citrobacter freundii* (*n* = 4), and *Shigella boydii* (*n* = 5).

### 3.3. Characterization of ESBL Genes

The sequence analysis of the ESBL genes (*bla*_CTXM_, *bla*_TEM_, and *bla*_OXA_) of the isolated strains from different fish species showed the dominance of *bla*_CTX-M-1_ in the *Enterobacteriaceae* isolates. The ESBL genes were (species/number of isolates) *bla*_CTX-M-1_ for *E. coli*/15, *K. pneumonia*/8, *C. freundii*/1, and *Sh. boydii*/1; *bla*_CTX-M-1_+ *bla*_OXA-1_ for *E. coli*/4 and *K. pneumonia*/3; *bla*_CTX-M-1_+ *bla*_TEM-1-a_ for *K. pneumonia*/2; *bla*_CTX-M-15_+ *bla*_TEM-1-a_ for *K. pneumonia*/1 and *Sh. boydii*/1; *bla*_CTX-M-15_+ *bla*_OXA-1_ for *K. pneumonia*/1; *bla*_CTX-M-15_ for *E. coli*/3, *K. pneumonia*/1, and *Sh. boydii*/3; and *bla*_CTX-M-9_ for *C. freundii*/3.

### 3.4. Resistance Mechanism of Non-*β*-Lactam Antimicrobial Agents

Resistance to the sulfonamide of ESBL-Eb isolates (*n* = 43) was shown to be conferred by either *sul 1* genes for 15 isolates (*E. coli* [*n* = 7], *K. pneumonia* [*n* = 4], *C. freundii* [*n* = 3], and *Sh. boydii* [*n* = 1]) or *sul 2* genes for 13 other strains (*E. coli* [*n* = 10], *K. pneumonia* [*n* = 2], and *C. freundii* [*n* = 1]), with no *sul* gene being detected for the last 15 strains. For the resistance to the tetracycline, the *tet A* gene appeared in all 22 resistant isolates of *E. coli* (*n* = 21) and *K. pneumonia* (*n* = 1). Besides, for quinolone family resistance, only six out of the 22 strains harbored the aac(6′)-Ib*-cr* gene for *E. coli* (*n* = 5) and *K. pneumonia* (*n* = 1). Analysis of *qnr* plasmids by PCR allowed the detection of the *qnrA* gene in *E. coli* isolates (*n* = 10) and the *qnrB* gene in 10 strains [*E. coli* (*n* = 9) and *K. pneumonia* (*n* = 1)].

### 3.5. Phylogenetic Groups of *E. coli* Isolates and Virulence Factors

Amplification of the three genes *ChuA*, *YjaA*, and *TSPE4C2*and their presence or absence in 24 ESBL-producing *E. coli* strains has revealed that 20 strains (83.3%) appeared to belong to the group D and 2 strains (8.3%) to the group B2, while the two other strains (8.3%) belonged to the group A. Characterization of the virulence factor demonstrated the prevalence of the *fimA* gene in nine *E. coli* isolates (37.5%), but there was no strain revealing the three other virulence factors considered (*eae*, *aer*, and *cnf1*) (Tables 2 and 3).

## 4. Discussion

In the present study, 59 Gram-negative and cefotaxime resistant isolates, belonging to the *Enterobacteriaceae* family, were recovered out of 126 samples of different wild fish species, collected from the lagoon of Bizerte. Molecular identification showed the dominance of *E. coli* isolates (40%) along with *K. pneumoniae* (*n* = 21), *C. freundii* (*n* = 8), and *Sh. boydii* (*n* = 6). The high frequency of *E. coli* and *K. pneumoniae* isolation of 40.7 and 35.6%, respectively, registered in this study is well reported in several similar studies worldwide and different environments [29–32].

In addition, the detection of CTX-resistant enterobacteria strains in the gills and the stomach contents of the different fish species was realized. Thus, the gill content appeared to show higher frequency isolation of bacteria resistant to cefotaxime as compared to those from the stomach content. This would infer that the gills may be a very favorable reservoir to host resistant flora as compared to the stomach. Resistant *Enterobacteriaceae* in the gills seems to be caused by the ingestion of water contaminated with fecal bacteria and antibiotic residues in the lagoon of Bizerte. Some studies reported that the high frequency of concentrations of antibiotics reported in the marine environment and their potential impacts on the aquatic ecosystems explain the widespread of antibiotic resistance largely reported in fish, marine mammals, and seabirds living in coastal waters [33].

In addition, the most important resistance frequencies, registered in the different fish species tested, were shown for *Sarpa salpa*, followed by the species of *Trachurus trachurus*, *Chelon labrosus*, *Mullus surmuletus*, and, finally, *Dicentrarchus labrax*; such frequencies showed ratios fluctuating between 1.6 and 0.4. However, reports on bacteria isolation were low variable and being closer to zero for *Mugil cephalus*, *Lithognathus mormyrus*, *Pomatomus saltatrix*, and *Solea solea*. These reports infer a great chance of fish exposure to bacteria resistant to cefotaxime and other antibiotics, and such exposure seems to be primarily related to the biological behavior of the fish as well as their wide geographical distribution in the marine environment and lagoons [34–37].

But, we have detected 47 ESBL-Eb-producing isolates, especially in *E. coli* (*n* = 22), *K. pneumoniae* (*n* = 16), *Sh. Boydii* (*n* = 5), and *C. freundii* (*n* = 4). This agrees with the recent report of Ben Said et al. [38] that has described ESBL *Enterobacteriacea*e isolates in sewage water, in Tunisia. Singh et al. [39] have also reported a study of multiple antibiotic-resistant, ESBL-producing enterobacteria in fresh seafood such as *Escherichia coli* the predominant species followed by *Klebsiella oxytoca* and *K. pneumonia*. It was shown that the ESBL-positive phenotype is detected in 169 (78.60%) tested isolates, with *E. coli* being the predominant species (53), followed by *Klebsiella oxytoca* (27) and *K. pneumoniae* (23).

Characterization of the ESBL genetic profile by amplification and sequencing of different CTX-M groups showed the dominance of *bla*_CTX-M-1_ among 35 isolates of *E. coli* (*n* = 19), *K. pneumonia* (*n* = 13), *Sh. Boydii* (*n* = 6), and *C. freundii* (*n* = 8). A similar study conducted, in Tunisia, by Ben Said et al. [40] revealed the detection of enterobacteria-producing ESBL species in agronomic soil, irrigation water, and various vegetables. Besides the prevalence of CTX-M-1 enzymes, CTX-M-15 (*n* = 10) and CTX-M-9 (*n* = 3) were also detected in the study. Among these strains, we have noticed the existence of an association between *bla*_CTX-M-1_/*bla*_CTX-M-15_ and *bla*_OXA-1_ and *bla*_TEM-1a_. Indeed, the association between genes encoding cefotaximases and those of other beta-lactamases (TEM and OXA types) has been frequently identified among our isolates and also previously reported in others [41].

All these results confirmed the high circulation of these resistance genes in the environment and the lagoon of Bizerte. In addition, the gene *bla*_CTX-M-15_, known as an ESBL gene usually associated with strains found in the environment, and the gene *bla*_CTX-M-1_, known as the most detected ESBL gene in food products, in vegetation, and in different natural environments, explained the origin of the high transmission and dissemination of these variants of resistant bacteria through the residual releases into the lagoon of Bizerte.

It is well confirmed that the environment could play a very important role in the diffusion of resistance. Antibiotics used in agriculture and fish farming or arboriculture are frequently found as metabolites in the soils and the waters where they exert and maintain high genetic selection pressure [40].

Most of our ESBL-Eb have shown a multiresistance phenotype and carried a different type of resistance genes (*tet*, *sul*) encoding for tetracycline and sulfonamide resistance, respectively, with this character being typical of ESBL-producing bacteria in other studies [40, 42].

For quinolone resistance, it was conferred by aac(6′)-ib*-cr*, *qnrA*, *and qnrB* genes. These antibiotic genetic determinants are specifically related to pathogenic clinical strains and were recently detected in the environment [40, 43, 44].

Detection of quinolone plasmid QNR confirmed the circulation of these resistance genes by mobile genetic elements. In parallel, determining the phylogenetic groups and analyzing the different virulence factors of *E. coli* isolates resistant to cefotaxime showed mainly the dominance of the phylogenetic group D with 81.9% (18/22 isolates). This group is known to be principally composed of pathogenic strains called “extraintestinal” pathogens. Among other antibiotic-resistant isolates, two strains (9%) belonged to the phylogenetic group B2. This group B2 is known to be the most virulent strain group [41]. Finally, only two strains belonged to the phylogenetic group A as known as “commensal” phylogenetic group strains. All these results are similar to those reported by Ben Said et al. [38]. The investigation on the 4 known virulence genes studied in all *E. coli* isolates has revealed the dominance of the *fimA* gene that belongs to the phylogenetic groups D and B2. The study of Jouini et al. [45] has confirmed these findings and has found that all strains isolated from many varieties of food presented the *fimA* gene of virulence. Therefore, no other isolates have revealed at least one of the four genes investigated in the present work; these strains could host other virulence determinants not yet investigated.

## 5. Conclusion

Our study appeared to be the first work done in Tunisia concerning the lagoon of Bizerte and showed a high occurrence of ESBL-*Enterobacteriaceae* as well as the CTX-M-1 group, in some tested wild fish species. It allowed determination of *tet*, *sul*, aac(6′)-ib-*cr*, and *qnr* resistance genes that confer resistance to tetracycline, sulfonamide, and quinolone, respectively. These findings demonstrated the role of the Bizerte lagoon as hotspot collectors of ESBL-*Enterobacteriaceae* with high likelihood of dissemination and spread to humans and animals throughout the food chain.

## Figures and Tables

**Table 1:** Distribution of positive bacterial strains resistant to cefotaxime according to the different species of fish sampled.

Family	Common name	Scientific name	Number of samples	Distribution isolates	
				Gills	Stomach contents
Moronidae	Loup	*Dicentrarchus labrax*	14	4	2
Carangidae	Chinchard commun	*Trachurus trachurus*	14	6	6
Mugilidae	Muges	*Mugil cephalus*	14	2	0
	Mullet	*Chelon labrosus*	14	5	3
Mullidae	Rouget de roche	*Mullus surmuletus*	14	5	3
Pomatomidae	Tassergal	*Pomatomus saltatrix*	14	0	0
Soleidae	Sole commune	*Solea solea*	14	0	0
Sparidae	Marbré	*Lithognathus mormyrus*	14	1	0
	Saupe	*Sarpa salpa*	14	12	10
Total			126	35	24

**Table 2:** Characteristic of ESBL-Eb strains detected in fish from lagoon of Bizerte.

Isolates	Sampling	Fish species	ESBL	Resistance to non-*β*-lactam	Resistance genes	Phylogenetic group	Virulence factor
EC 138	SC	*S. salpa*	*CTX-M-1+OXA-1*	TET, NAL, SUL	*tet A*, *sul 2*, *qnrB*	*D*	*—*
EC 155	G	*T. trachurus*	*CTX-M-1+OXA-1*	TET, NAL, SUL	*tet A*, *sul 1*, *qnrB*	*D*	*—*
EC 159	SC	*T. trachurus*	*CTX-M-1+OXA-1*	TET, NAL, SUL	*tet A*, *sul 2*, *qnrB*	*D*	*—*
EC 163	G	*T. trachurus*	*CTX-M-1+OXA-1*	TET, NAL, SUL	*tet A*, *sul 2*, *qnrB*	*D*	*—*
EC 71	SC	*S. salpa*	*CTX-M-1*	TET, NAL, SUL	*tet A*, *sul 2*, *qnrB*	*B2*	*fimA*
EC 80	G	*S. salpa*	*CTX-M-1*	TET, NAL, SUL	*tet A*, *sul 1*, *aac(6*′*)ib-cr*	*D*	*fimA*
EC 87	SC	*S. salpa*	*CTX-M-1*	TET, NAL, SUL	*tet A*, *sul 2*, *qnrB*	*D*	*fimA*
EC 94	SC	*S. salpa*	*CTX-M-1*	TET, NAL, SUL	*tet A*, *sul 2*, *aac(6*′*)ib-cr*	*D*	*—*
EC 98	G	*S. salpa*	*CTX-M-1*	TET, NAL, SUL	*tet A*, *sul 1*, *qnrA*	*B2*	*fimA*
EC 101	SC	*S. salpa*	*CTX-M-1*	TET, NAL, SUL	*tet A*, *qnrA*	*D*	*fimA*
EC 105	G	*S. salpa*	*CTX-M-1*	TET, NAL, SUL	*tet A*, *sul 1*, *aac(6*′*)ib-cr*	*D*	*fimA*
EC 109	SC	*S. salpa*	*CTX-M-1*	TET, NAL, SUL	*tet A*, *sul 2*, *qnrA*	*D*	*—*
EC 113	G	*S. salpa*	*CTX-M-1*	TET, NAL, SUL	*tet A*, *sul 2*, *qnrA*	*D*	*—*
EC 119	G	*S. salpa*	*CTX-M-1*	TET, NAL, SUL	*tet A*, *sul 1*, *qnrA*	*D*	*—*
EC 129	G	*S. salpa*	*CTX-M-1*	TET, NAL, SUL	*tet A*, *sul 2*, *aac(6*′*)ib-cr*, *qnrB*	*D*	*—*
EC 131	G	*S. salpa*	*CTX-M-1*	TET, NAL, SUL	*tet A*, *sul 1*, *qnrB*	*D*	*—*
EC 182	G	*P. saltatrix*	*CTX-M-1*	TET, STR	*tet A*	*D*	*—*
EC 319	SC	*M. surmuletus*	*CTX-M-1*	CN, TOB, SUL	*—*	*A*	*—*
EC 61	SC	*S. salpa*	*CTX-M-15*	TET, NAL, SUL	*tet A*, *qnrA*	*D*	*fimA*
EC 62	G	*S. salpa*	*CTX-M-15*	TET, NAL, SUL	*tet A*, *sul 2*, *aac(6*′*)ib-cr*	*D*	*fimA*
EC 68	G	*S. salpa*	*CTX-M-15*	TET, NAL, SUL	*tet A*, *sul 1*, *qnrB*	*D*	*fimA*
K. p 286	SC	*T. trachurus*	*CTX-M-1+TEM-1-a*	CN, TOB, SUL	*—*		
K. p 291	G	*T. trachurus*	*CTX-M-1+TEM-1-a*	CN, TOB, SUL	*—*		
K. p 282	G	*T. trachurus*	*CTX-M-15+TEM-1-a*	CN, TOB, SUL, STR	*—*		
K. p 135	SC	*S. salpa*	*CTX-M-1+OXA-1*	TET, NAL, SUL	*tet A*, *sul 1*, *aac(*6′*)ib*		
K. p 206	G	*D. labrax*	*CTX-M-1+OXA-1*	CN, TOB	*—*		
K. p 246	SC	*Ch. labrosus*	*CTX-M-1+OXA-1*	CN, TOB	*—*		
K. p 216	G	*D. labrax*	*CTX-M-15+OXA-1*	CN, TOB	*—*		
K. p 211	SC	*D. labrax*	*CTX-M-1*	CN, TOB	*—*		
K. p 222	SC	*D. labrax*	*CTX-M-1*	CN, TOB, SUL	*—*		
K. p 234	G	*Ch. labrosus*	*CTX-M-1*	C, CN, TOB	*—*		
K. p 243	G	*Ch. labrosus*	*CTX-M-1*	CN, TOB, SUL	*sul 2*		
K. p 249	G	*Ch. labrosus*	*CTX-M-1*	CN, TOB, SUL	*sul 2*		
K. p 254	SC	*Ch. labrosus*	*CTX-M-1*	CN, TOB, SUL	*sul 1*		
K. p 257	G	*Ch. labrosus*	*CTX-M-1*	CN, TOB, SUL	*—*		
K. p 263	SC	*T. trachurus*	*CTX-M-1*	CN, TOB, SUL	*—*		
K. p 219	G	*D. labrax*	*CTX-M-15*	CN, TOB	*—*		
C342	G	*T. trachurus*	*CTX-M-1*	CN, TOB, SUL	*sul 2*		
C66	SC	*S. salpa*	*CTX-M-9*	—	*—*		
C79	SC	*S. salpa*	*CTX-M-9*	—	*—*		
C225	G	*D. labrax*	*CTX-M-9*	SUL	*sul 1*		
SH278	SC	*T. trachurus*	*CTX-M-15+TEM-1-a*	CN, TOB, SUL	*—*		
SH260	SC	*Ch. labrosus*	*CTX-M-1*	CN, TOB, SUL	*—*		
SH273	G	*T. trachurus*	*CTX-M-15*	CN, TOB, SUL	*—*		
SH307	G	*M. surmuletus*	*CTX-M-15*	CN, TOB, SUL	*—*		
SH315	G	*M. surmuletus*	*CTX-M-15*	CN, TOB	*—*		

SC: stomach content analysis; G: gills; EC: *Escherichia coli*; K. p: *Klebsiella pneumoniae*; CF: *Citrobacter freundii*; (SH): *Shigella boydii*; T: Thermococcus; *S. salpa*: *Sarpa salpa*; *T. trachurus*: *Trachurus trachurus*; *P. saltatrix*: *Pomatomus saltatrix*; *M. surmuletus*: *Mullus surmuletus*; *D. labrax*: *Dicentrarchus labrax*; *Ch. labrosus*: *Chelon labrosus*; TET: tetracycline; Nal: nalidixic acid; TOB: tobramycin; SUL: sulfonamide; CN: gentamicin; STR; streptomycin; C: chloramphenicol.

**Table 3:** Characteristic of non ESBL-Eb strains detected in fish from lagoon of Bizerte.

Isolates	Sampling	Fish species	Resistance to non-*β*-lactam	Resistance genes	Phylogenetic group	Virulence factor
EC 3	G	*S. salpa*	CIP, TET, NAL	*tet A*, *qnrB*	*D*	*—*
EC 24 (1)	G	*L. mormyrus*	—	*—*	*D*	*—*
K. p 25	G	*S. salpa*	NAL	*qnrB*		
K. p 75	G	*S. salpa*	—	*—*		
K. p 296	SC	*M. surmuletus*	TOB, SUL	*sul 1*		
K. p 311	SC	*M. surmuletus*	TOB, SUL	*sul 1*		
K. p348	G	*M. surmuletus*	STR	*—*		
C170	G	*P. saltatrix*	—	*—*		
C145	SC	*T. trachurus*	—	*—*		
C242	G	*Ch. labrosus*	CN, TOB, SUL	*sul 1*		
C270	SC	*T. trachurus*	CIP, TOB, SUL	*sul 1*		
SH300	G	*M. surmuletus*	TOB, SUL	*sul 1*		

SC: stomach content analysis; G: gills; EC: *Escherichia coli*; K. p: *Klebsiella pneumoniae*; CF: *Citrobacter freundii*; SH: *Shigella boydii*; *S. salpa*: *Sarpa salpa*; *T. trachurus*: *Trachurus trachurus*; *P. saltatrix*: *Pomatomus saltatrix*; *M. surmuletus*: *Mullus surmuletus*; *Ch. labrosus*: *Chelon labrosus*; *L. mormyrus*: *Lithognathus mormyrus*; TET: tetracycline; Nal: nalidixic acid; TOB: tobramycin; SUL: sulfonamide; CN: gentamicin; STR; streptomycin; C: chloramphenicol.

## Data Availability

All data used to support the findings of the study are approved and included in the article.
